# In Vitro Antiproliferative Apoptosis Induction and Cell Cycle Arrest Potential of Saudi Sidr Honey against Colorectal Cancer

**DOI:** 10.3390/nu15153448

**Published:** 2023-08-04

**Authors:** Husam Qanash, Abdulrahman S. Bazaid, Naif K. Binsaleh, Mitesh Patel, Omar W. Althomali, Bodor Bin Sheeha

**Affiliations:** 1 Department of Medical Laboratory Science, College of Applied Medical Sciences, University of Ha’il, Hail 55476, Saudi Arabia; n.binsaleh@uoh.edu.sa; 2 Department of Biotechnology, Parul Institute of Applied Sciences and Centre of Research for Development, Parul University, Vadodara 391760, Gujarat, India; patelmeet15@gmail.com; 3 Department of Physiotherapy, College of Applied Medical Sciences, University of Ha’il, Hail 55476, Saudi Arabia; o.althomali@uoh.edu.sa; 4 Department of Rehabilitation Sciences, College of Health and Rehabilitation Sciences, Princess Nourah Bint Abdulrahman University, P.O. Box 84428, Riyadh 11671, Saudi Arabia; bhbinsheeha@pnu.edu.sa

**Keywords:** Saudi Sidr honey, anticancer, antiproliferative apoptosis, natural product, colorectal cancer

## Abstract

A range of natural products have been extensively studied for their chemopreventive potential for cancer, including those that inhibit growth and induce apoptosis. Sidr honey derived from the *Ziziphus* or *Lote* tree (*Ziziphus spina-christi*, *Ziziphus lotus*, or *Ziziphus jujuba*) is used in a wide range of traditional medicine practices. In the current study, the Saudi Sidr honey was analyzed by means of a GC–MS chromatogram and investigated for its antiproliferative effects on colorectal cancer cells (HCT-116), breast cancer cells (MCF-7), and lung cancer cells (A-549), as well as its apoptosis induction and cell cycle arrest potentials against human colorectal cancer cells (HCT-116). The effects of Saudi Sidr honey on cells were determined using the MTT assay and the clonogenic assay. The induction of apoptosis was studied using Annexin V-FITC flow cytometry analysis. The propidium iodide staining method was used to detect cell cycle arrest via flow cytometry. By means of performing GS–MS and HR-LCMS analysis, 23 different chemical components were identified from Saudi Sidr honey. A dose–response analysis showed that Saudi Sidr honey was more effective against HCT-116 (IC_50_ = 61.89 ± 1.89 µg/mL) than against MCF-7 (IC_50_ = 78.79 ± 1.37 µg/mL) and A-549 (IC_50_ = 94.99 ± 1.44 µg/mL). The antiproliferation activity of Saudi Sidr honey has been found to be linked to the aggregation of cells during the G1 phase, an increase in early and late apoptosis, and necrotic cell death in HCT-116 cells. Considering these promising findings that highlight the potential use of Saudi Sidr honey as an antitumor agent, further research should be carried out with the aim of isolating, characterizing, and evaluating the bioactive compounds involved in Sidr honey’s antiproliferative activity to better understand the mechanism of their action.

## 1. Introduction

Cancer is a leading cause of morbidity and mortality worldwide [1]. There are over ten million cancer cases diagnosed each year throughout the world, and more than half of patients die from it [2,3]. Cancer ranks as the second leading cause of death in many countries after cardiovascular diseases [4,5]. Colorectal cancer (CRC) is a type of cancer that affects the colon (large intestine) and rectum [6]. According to the World Health Organization, it is the third most common cancer in men (after lung and prostate cancers) and the second most common cancer in women (after breast cancer) [7]. In 2020, there were an estimated 1.9 million new cases of colorectal cancer that led to 935,000 deaths globally [1]. CRC often develops slowly over a period of years, beginning as a benign growth called a polyp. However, if left untreated, these polyps can become cancerous and spread to other parts of the body [8]. Symptoms of colorectal cancer may include changes in bowel habits, such as diarrhea or constipation; blood in the stool; abdominal pain; and unexplained weight loss. However, some people with CRC may not experience any symptoms, especially in the early stages of the disease [7].

A cancerous tumor develops when there is deregulation of any mechanisms that are involved in, or that are necessary to maintain, normal cell growth and proliferation, such as cell division and apoptosis [9,10]. To develop anticancer drugs, it is crucial to identify the different regulatory mechanisms in cancer cells that are responsible for transformation in order to target those mechanisms specifically [11,12,13]. Currently, treatment options for CRC depend on several factors including the location of the tumor, the stage of the disease, and the patient’s overall health. Treatment may include surgery to remove the cancerous tissue; the use of several drugs such as fluoropyrimidine, irinotecan, oxaliplatin, and capecitabine; radiation therapy; chemotherapy; or a combination of these approaches [14].

On the other hand, CRC patients face several side effects associated with these treatment options [15]. Therefore, scientists are seeking new drugs that would have minimal toxicity to normal or non-cancerous cells and actively reduce cancer development [16,17]. There are a vast variety of bioactive metabolites that are found in natural products and exhibit anticancer activity and low toxicity. This versatility makes natural products a perfect source for new drug candidates against diseases including cancer [18].

Honey is a natural sweet substance produced by honey bees from the nectar of flowers [19]. It has been used for medicinal purposes for thousands of years and has recently gained attention for its potential anticancer activity [20,21,22,23]. Honey is a complex mixture of various compounds, including sugars, organic acids, flavonoids, and phenolic acids, which have been shown to possess a variety of therapeutic properties, including antioxidant, antimicrobial, anti-inflammatory, and anticancer effects [24,25,26,27]. Saudi Arabia is known for its diverse range of honeys, each with its unique characteristics and flavours. For example, Acacia honey, renowned for its mild taste and high fructose content; Sumar honey derived from wildflowers with distinct medicinal properties; and Talh honey extracted from the nectar of palm trees, offering a rich, caramel-like flavour. However, among these, Saudi Sidr honey stands out for several reasons. Its exclusivity, derived from the rare Sidr tree, contributes to its desirability. Its distinctive and robust taste, coupled with its potential medicinal properties, contributes to its popularity [28,29].

Some studies have shown that honey can induce apoptosis (programmed cell death) and inhibit the growth and proliferation of cancer cells [23,30,31,32]. While more research is needed to fully understand the mechanisms behind honey anticancer properties and to determine the optimal dose and delivery methods, the potential of honey as a complementary or alternative therapy for cancer is an exciting area of study. Therefore, the present study aimed to evaluate the antiproliferative effects and the apoptosis- and cell cycle arrest-inducing effects of Saudi Sidr honey in colorectal cancer cells (HCT-116).

## 2. Materials and Methods

### 2.1. Collection of Honey Sample

**Figure 1:**
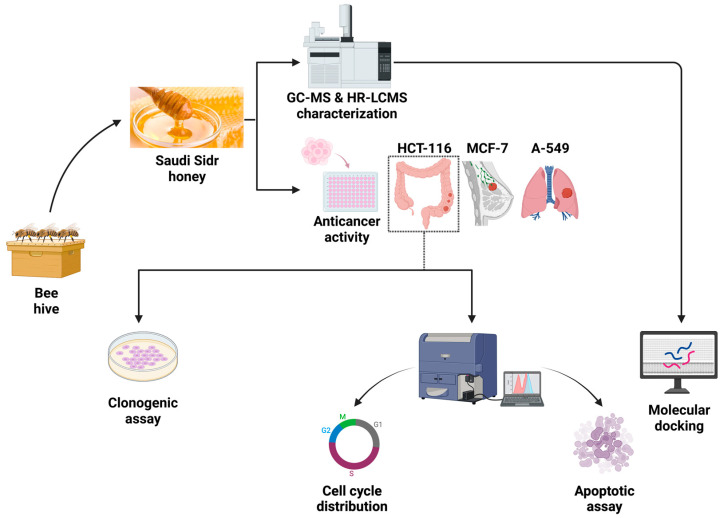
Image illustrating the Saudi Sidr honey, which was exposed to various appropriate assays to identify the chemical components of the honey and investigate its anticancer activities.

The Sidr honey sample that was investigated in this study because of its traditional and medicinal applications [33] was purchased in January 2022 from a local market in Al Baha, Saudi Arabia. The sample was immediately transferred to the laboratory and began being processed. There were no signs of granulation, fermentation, or contamination in the honey. The honey was maintained at a normal laboratory temperature throughout the study and several experiments were performed on it (Figure 1).

### 2.2. Phytochemical Analysis of Saudi Sidr Honey

A Shimadzu Nexis GC-2030 and a QP2020 NX MS were used to conduct a gas chromatography–mass spectrometry (GC–MS) analysis to assess the composition of Saudi Sidr honey. The material was separated using the SH-Rxi-5Sil (30 m, 0.25 mm ID, 0.25 m df, Shimadzu) column. The temperature was raised to 270 °C for the final separation after being brought to 50 °C for three minutes then increased by 5 °C every minute until 250 °C was reached. Helium was employed as a carrier gas to transport the 20 µL of sample that was added to the system. An analysis of volatile compounds was carried out in the electron impact ionization mode at 70 eV, and the mass range was *m*/*z* 35~500. The interface and source temperatures were 200 °C. To ascertain the probable composition of the honey, the peaks produced from the GC–MS separation were compared to the NIST database [34]. The LabSolutions DB GCMS software (version 4.52) (Shimadzu Corporation, Kyoto, Japan) was utilized for conducting the GC–MS analysis. Apart from GC–MS analysis, high resolution liquid chromatograph mass spectrometer (HR-LCMS) analysis was also carried out to detect the presence of different bioactive metabolites using a UHPLC-PDA detector mass spectrophotometer (HR-LCMS 1290 Infinity UHPLC System), Agilent Technologies^®^, Santa Clara, CA, USA. In the liquid chromatographic system, there was a HiP sampler, a binary gradient solvent pump, a column compartment, and a quadrupole time-of-flight mass spectrometer (MS Q-TOF) equipped with two Agilent Jet Stream Electrospray (AJS ES) ion sources. As part of the separation process, 10 µL of sample was injected into the system, followed by the separation of the sample in an SBC18 column (2.1 × 50 mm, 1.8 μm particle size). The solvents A and B were 1% formic acid in deionized water and acetonitrile, respectively. The flow rate was 0.350 mL/min, and the MS detection was performed with a Q-TOF MS. Mass spectra of the compounds were compared to their unique fragmentation patterns in order to identify them. It was made possible to identify the bioactive constituents by using Compound Discoverer 2.1, ChemSpider, and PubChem as the main tools for the identification of compounds.

### 2.3. Cell Culture

Culturing of three cancer cell lines including human colorectal cancer (HCT-116), breast cancer (MCF-7), and lung cancer (A-549) was performed using Dulbecco’s Modified Eagle Medium (DMEM) supplemented with 10% fetal bovine serum (FBS) (Hi-Media^®^, Mumbai, India), 10,000 U/mL penicillin, and 5 mg/mL of streptomycin (Hi-Media^®^, Mumbai, India). To maintain them in their optimum state, the cells were kept at 37 °C in a humidified atmosphere of 5% CO_2_ [35].

### 2.4. Cell Viability Assay

To determine the viability of all three cancer cells, Saudi Sidr honey was tested using the MTT assay. Cells were harvested from T-25 flasks by trypsinizing them and aspirating them into a 5 mL centrifuge tube. Centrifugation at 3000 rpm was used to collect the cells. Culture medium was used to adjust the cell count so that approximately 10,000 cells were suspended in 200 µL. The wells of the 96-microtiter plate were filled with 200 µL of cell suspension, and the plate was then incubated at 37 °C in 5% CO_2_ for 24 h. At the end of incubation, the spent medium was removed, and the cells were treated with 200 µL of different concentrations of Saudi Sidr honey (0, 2, 4, 6, 8, 10%) and incubated for 24 h at 37 °C under 5% CO_2_ conditions. Afterwards, 200 µL of fresh medium with 10% MTT reagent was poured into each well and further incubated for 3 h at 37 °C under 5% CO_2_ conditions. Then, developed formazan crystals were dissolved by means of gentle agitation using a gyratory shaker with 100 µL of DMSO. Finally, absorbances at 570 nm and 630 nm were measured using a microplate reader. The dose–response curve was prepared to calculate the amount of drug needed to inhibit 50% (IC_50_) of the cell growth after subtracting the background and blank values [36,37].

### 2.5. Colony Formation Assay

For the purpose of assessing the antineoplastic effects of Saudi Sidr honey on in vitro cell proliferation, the clonogenic assay was performed as described previously [38]. Briefly, cells were cultured in T-25 tissue culture flasks for 24 h and treated with Saudi Sidr honey for 24 h. Afterwards, the cells were trypsinized and then plated into six-well plates (per well, 200 cells/2 mL medium) and grown for 8 days. For the purpose of fixing the colonies, methanol was used as a fixative. To stain the colonies, crystal violet (0.4 g/L) was used as a stain, which was then photographed, analyzed, and counted using the ImageJ software (v1.48).

### 2.6. Annexin V–PI Apoptosis Assay

The cells were grown in 6-well plates as described above. The spent medium was removed from the wells, and 1 mL of PBS was added to wash the cells. As a next step, the Saudi Sidr honey (IC_50_) was added to 2 mL of culture medium, and the cells were treated overnight at 37 °C in a CO_2_ incubator. The wells that were left untreated were used as the negative control. After the incubation, the cells from each well were collected and transferred to a centrifuge tube to collect the cells by performing centrifugation at 3000 rpm for 5 min. The obtained cell pellet was washed twice with 500 µL of PBS. Then, the cells were resuspended in 1X binding buffer at a concentration of 1 × 10^6^ cells/mL. Then, the cells were divided into groups of control, unstained cells, Annexin only, PI only, and treatment. The Annexin V-FITC and PI were added to respective labelled tubes. After vertexing and incubating for 10 min at room temperature, 1X binding buffer was added to each tube and an analysis using a flow cytometer was performed within an hour [39].

### 2.7. Cell Cycle Analysis

As described above, HCT-116 cells were plated and treated with Saudi Sidr honey (IC_50_) for 24 h. Following the incubation period, the cells were harvested using trypsin. The cells were collected in 1.5 mL centrifuge tubes and washed once with 500 µL chilled PBS. To obtain a mono-dispersed cell suspension with minimal cell aggregation, approximately 1 × 10^6^ cells were suspended in 100 μL of PBS and vortexed gently. This suspension was then transferred to centrifuge tubes containing 70% ethanol on ice and incubated for 2 h to fix the cells. The cells were centrifuged after fixation and suspended in PI staining solution (0.1% (*v*/*v*) Triton X-100, 10 μg/mL PI and 100 μg/mL DNase-free RNaseA in PBS) and incubated in dark conditions for 30 min at room temperature. Then, flow cytometry was used to analyze the samples [40].

### 2.8. Molecular Docking Analysis

Molecular docking using AutoDock Vina was further performed to investigate the mechanisms of Saudi Sidr honey on anticancer activity [41]. The 3D structures of compounds in Saudi Sidr honey identified using GC–MS and HR-LCMS analysis were downloaded from the PubChem database. By using the Open Babel 3.1.1 tool, the three-dimensional (3D) structure of all the compounds was converted from .sdf to .pdb format. The Avogadro tool was used for energy minimization. The compound structure was minimized with MMFF94 force field. The steepest descent algorithm was used for optimization with a total of 5000 steps. During energy minimization, the structure was updated every 1 step and minimization was terminated when the energy difference was less than 0.1; it was then saved in .pdb format. From the RCSB-PDB database, 3D crystal structures were downloaded for proteins (CASP3—PDB ID: 1NME, BCL2—PDB ID: 4IEH, TP53—PDB ID: 3DCY). The crystal structure was modified by deleting water molecules. After that, hydrogen was added, and finally, Kollman charge was given to the protein structure. Protein coordinates were saved in .pdb format. The Open Babel tool was used again to convert all structure files from .pdb to .pdbqt format. PyMoL and Biovia Discovery Studio were used to analyze docked protein–ligand complexes [37].

### 2.9. Statistical Analysis

The MTT and colony formation assays were carried out in triplicate. The results were presented as mean ± SD of the number of experiments performed. The significance of the results was determined among the treatments using ordinary one-way ANOVA, followed by Bonferroni’s multiple comparisons test at *p* < 0.05. The analyses were carried out using the GraphPad Prism (Version 8.0.) software.

## 3. Results

### 3.1. Phytochemical Composition of Saudi Sidr Honey

**Figure 2:**
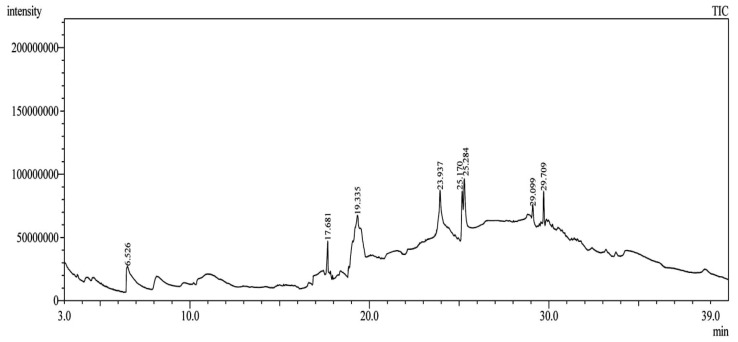
The analysis of Saudi Sidr honey by GC–MS chromatogram.

**Figure 3:**
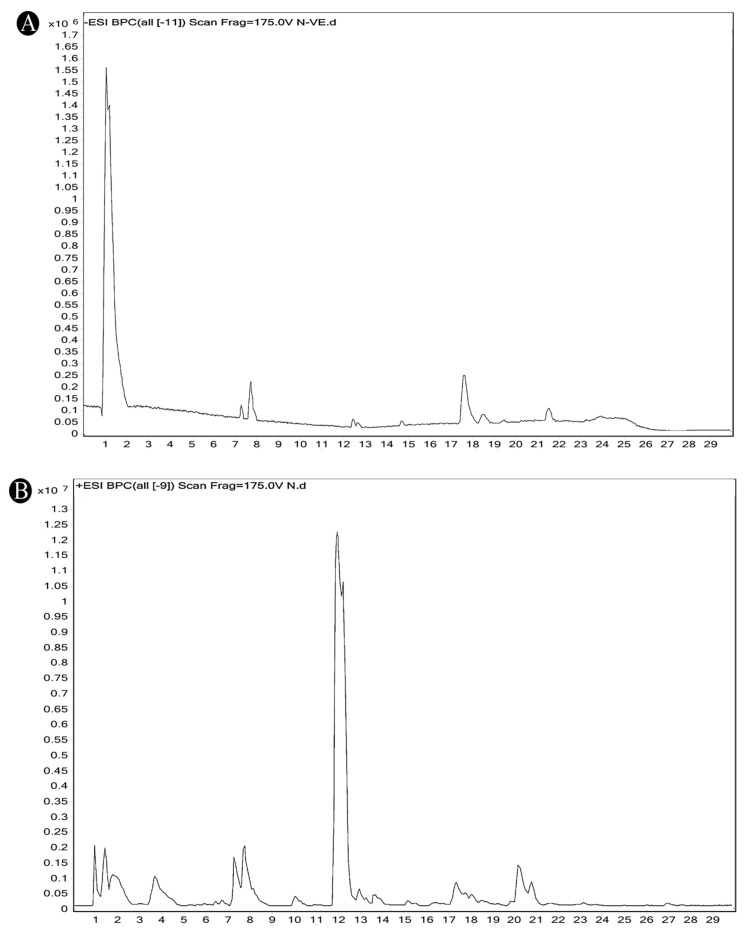
A chromatogram of Saudi Sidr honey obtained by performing HR-LCMS analysis. (**A**) Positive analysis. (**B**) Negative analysis.

Using gas chromatography–mass spectrometry (GC–MS), the Saudi Sidr honey was analyzed, and eight chemical components, based on their retention time (min), were identified (Table 1 and Figure 2). The highest retention times were recorded for 3,5-Dihydroxy-2-(3-methylbut-2-en-1-yl) (29.709 min) and 2,2,3,3-Tetramethylcyclopropanecarboxylic acid (29.099 min), followed by 1-Cyclohexylimidazolidin-2-one and 4-Butyl-3-methoxy-2-cyclopenten-1-one, where retention times of 25.284 and 25.170 min, respectively, were reported. However, Glyceraldehyde exhibited the lowest retention time of 6.526 min compared to other chemical constituents (Table 1 and Figure 2). On the other side, HR-LCMS analysis of the Saudi Sidr honey was performed using UHPLC-PDA-ESIMS/MS. The chromatograms were acquired both in negative and positive mode, and analysis was performed. As a result of the detailed UV spectra and mass spectra data as determined by the mass of the molecular ion peak and its fragments, as well as the neutral mass loss and known fragmentation patterns, in addition to comparisons with the available literature, different types of bioactive compounds were identified (Figure 3). The compounds, with their mass (*m*/*z*), retention time, and other information, were identified (Table 2).

### 3.2. Anticancer Activity of Saudi Sidr Honey

**Figure 4:**
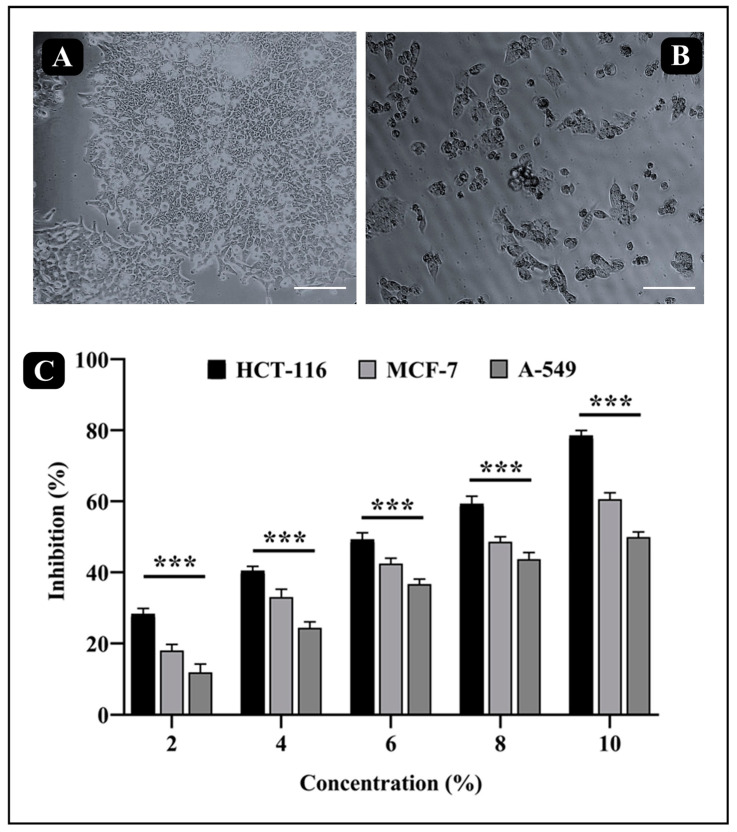
(**A**) Morphological analysis of HCT-116 cells under inverted microscope before treatment with Saudi Sidr honey. (**B**) Morphological analysis of HCT-116 cells under inverted microscope after 24 h treatment with Saudi Sidr honey (10%). (**C**) Cytotoxic activity of Saudi Sidr honey against various human cancer cell lines (HCT-116, MCF-7 and A-549) at different concentrations (%). Scale bar: 100 µm. Significance: *** *p* ≤ 0.001 with respect to the control—untreated cells of the respective cancer cell lines).

The MTT assay was performed to determine the anticancer potential of Sidr honey against HCT-116, MCF-7, and A-549 cancer cells. It was documented that Sidr honey had a higher anticancer activity against HCT-116 than against MCF-7 and A549 (Figure 4). It was found that Sidr honey had cytotoxic activity against HCT-116 cells in a dose-dependent manner. HCT-116 cells had an IC_50_ value of 61.89 ± 1.89 µg/mL, while MCF-7 and A-549 cells had IC_50_ values of 78.79 ± 1.37 µg/mL and 94.99 ± 1.44 µg/mL, respectively. Further assays against HCT-116 cells were carried out at IC_50_ concentration of Sidr honey.

### 3.3. Effect of Saudi Sidr Honey on Colony Formation of HCT-116 Cells

**Figure 5:**
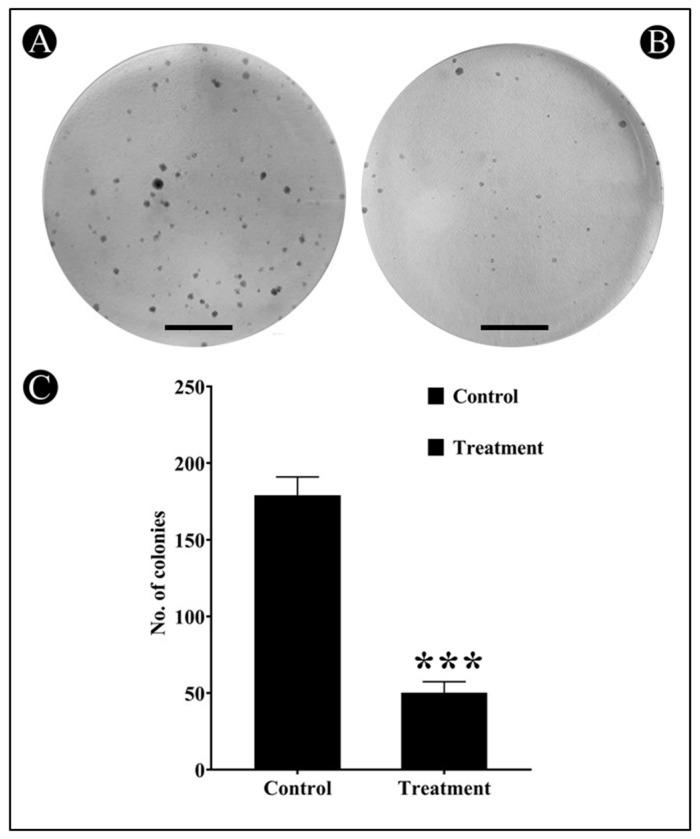
Clonogenic assay (crystal violate staining) in HCT-116 human colorectal cancer cell line exposed to the IC_50_ concentration of Saudi Sidr honey. (**A**) Untreated well of HCT-116 (control). (**B**) Treated-well represents a reduction in the number and size of colonies and was observed at 10×. Scale bar: 100 µm. (**C**) A bar graph representing the surviving fraction of HCT-116 cells in the absence and presence of Saudi Sidr honey. Significance: *** *p* ≤ 0.001 with respect to the control.

A colony formation assay was conducted to further investigate the anticancer potential of Sidr honey on HCT-116 cells. In general, cancer cells tend to grow in colonies when they are in contact with neighboring cells; if a cancer cell loses contact with neighboring cells, it dies. The results of clonogenic assays showed that treatment with Sidr honey significantly inhibited the colony formation of HCT-116 cells (Figure 5A–C). Crystal violet staining was used to calculate colonies. Compared to the control, Sidr honey at IC_50_ reduced the number of colonies by more than 50%. Using this assay, Sidr honey was further confirmed to have anticancer properties.

### 3.4. Apoptosis Induction by Saudi Sidr Honey

**Figure 6:**
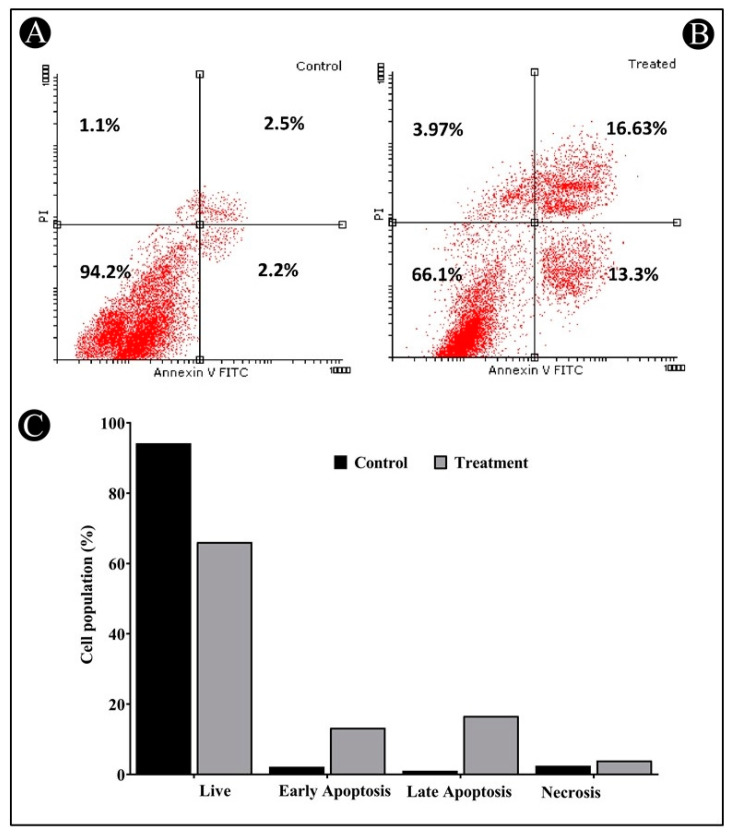
Annexin-V/PI apoptosis assay of untreated and treated HCT-116 human colorectal cancer cell line. (**A**) Untreated HCT-116 cells (control). (**B**) Distribution of treated HCT-116 with IC_50_ concentration of Saudi Sidr honey. (**C**) A bar graph of cell distribution in untreated and treated HCT-116 cells analyzed using flow cytometry.

Apoptosis is thought to begin with the translocation of phosphatidylserine (PS) from the inner to the outer membrane of the plasma membrane. A green fluorescence is produced when Annexin-V binds to PS when calcium ions are present. As a result of increasing membrane permeability during late apoptosis or necrosis, PI can also enter the cell and bind to DNA in the nucleus, thereby giving red fluorescence. Due to this, it is possible to distinguish between different cell populations in this assay, such as live cells, early apoptotic cells, late apoptotic/necrotic cells, and dead cells, by their labelling patterns. The Annexin-V/PI staining of HCT-116 cells after 24 h of incubation with Sidr honey revealed that 13.30% of the cells were in an early stage of apoptosis, 16.63% of the cells were in a late stage of apoptosis, and 3.97% of the cells were in a necrotic stage (Figure 6A–C). Therefore, this study clearly illustrates that Sidr honey was able to induce apoptosis in HCT-116 cells.

### 3.5. Saudi Sidr Honey Induces G1 Cell Cycle Arrest

**Figure 7:**
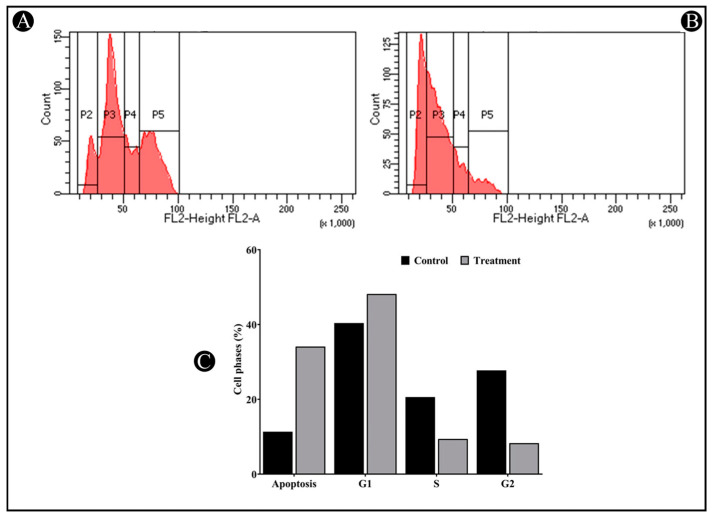
Cell cycle phase distribution for untreated and treated HCT-116 human colorectal cancer cell line with Saudi Sidr honey. (**A**) Untreated HCT-116 cells (control). (**B**) Saudi Sidr honey-treated HCT-116 cells. (**C**) A bar graph of average percentage of cells in different phases of the cell cycle in each treated and untreated group of HCT-116 cells.

The next step consisted of testing Sidr honey’s effect on the cell cycle of HCT-116 cells using flow cytometry. According to a cell cycle analysis, IC_50_ inhibited the G1 phase of the cell cycle. Sidr honey significantly increased G1 phase proportions in comparison to control cells while reducing S and G2M proportions (Figure 7A–C).

### 3.6. Molecular Docking Analysis

**Figure 8:**
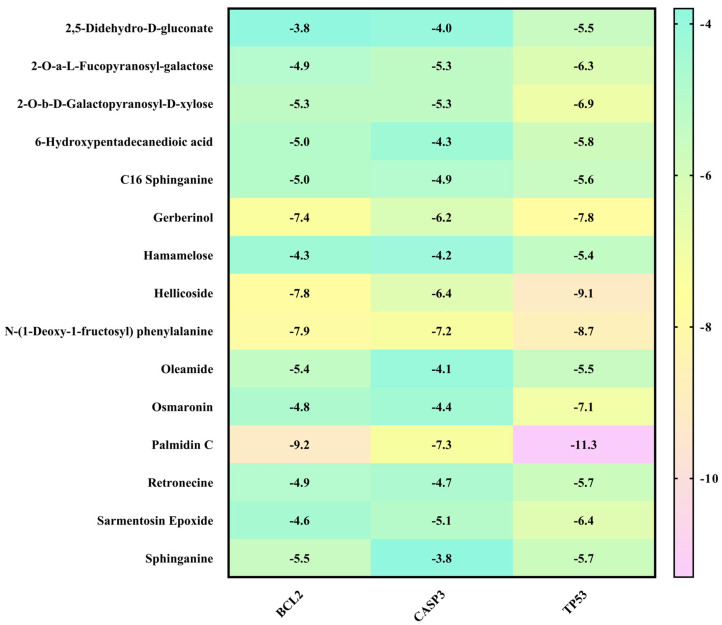
Binding affinities of top-rated pose of ligand–receptor complex.

**Figure 9:**
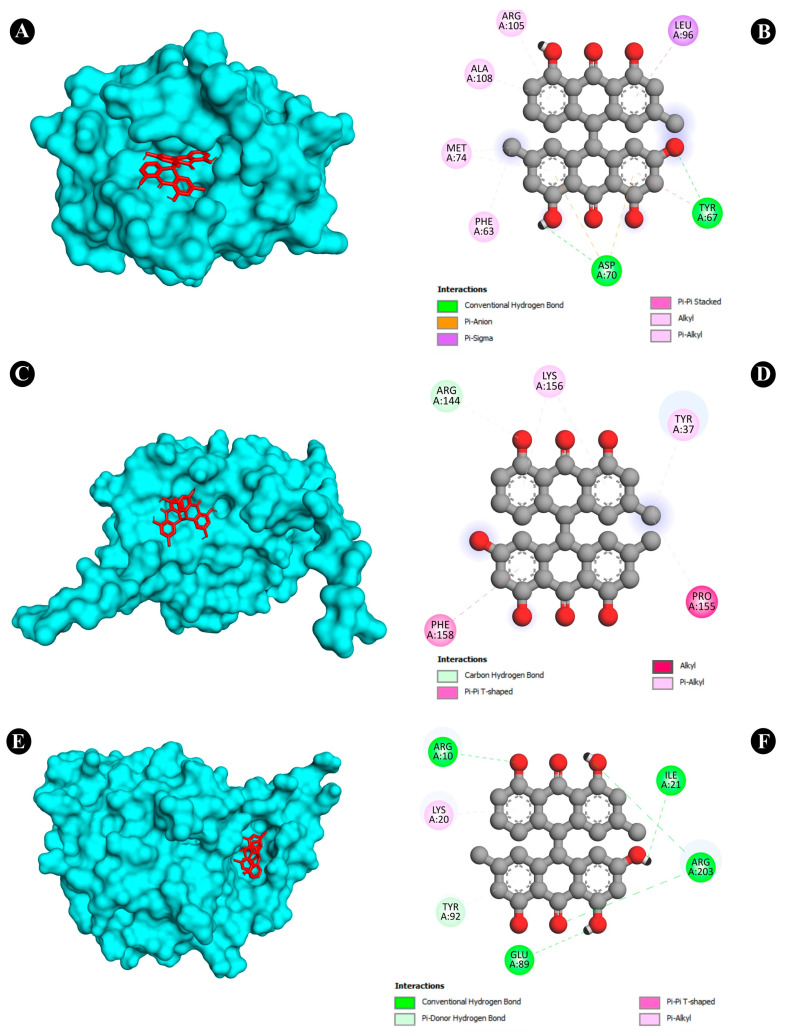
(**A**,**B**) Visualization of docking analysis of Palmidin C with BCL2. (**C**,**D**) Visualization of docking analysis of Palmidin C with CASP3. (**E**,**F**) Visualization of docking analysis of Palmidin C with P53. In A, C, and E, ligand is presented in red color, whereas protein is presented in cyan color.

To obtain a closer insight into the anticancer potential of Saudi Sidr honey compounds, molecular docking of IQ with the protein involved in apoptosis was performed. The parameters used in the molecular docking were first validated. Structure files (.pdb-format) of all compounds were docked separately against the receptor structures using the molecular docking software AutoDock 4.2.6. All the parameters used for the docking of all compounds with the receptors were kept the same except the grid center. The target proteins used for molecular docking (CASP3—PDB ID: 1NME, BCL2—PDB ID: 4IEH, TP53—PDB ID: 3DCY) were inside the grid box. AutoGrid was used for the preparation of the grid map using a grid box. The grid size was set to X = 57.993, Y = 54.798, and Z = 43.279 points for CASP3 receptor, for BCL2, X = 47.387, Y = 39.571, and Z = 38.496, and for TP53, X = 48.44, Y = 51.47, and Z = 54.09. Grid space was kept to 0.375 Å for all the receptors. The grid center for CASP3 was designated at dimensions (*x*, *y*, and *z*): 22.64, 100.80, and 11.06; for BCL2 at (*x*, *y*, and *z*): 6.24, 19.75, and 19.52; and for TP53 at (*x*, *y*, and *z*): 22.99, 37.34, and 3.71. The grid box was centered in such a way that it enclosed the entire binding site of both the receptors and provided enough space for the translation and rotation of ligands. The results of the molecular docking analysis revealed that the binding energy of the major identified bioactive compounds of Saudi Sidr honey was in the range of −3.1 to −11.3 kCal/mole. Regarding the binding energies, Palmidin C exhibited the highest binding affinities with all proteins (Figure 8). Palmidin C was observed occupying the active sites of different proteins in different ways (Figure 9A–F).

## 4. Discussion

Honey has been used for therapeutic purposes for centuries, and its potential as a natural remedy continues to be recognized. The unique chemical composition of honey gives it a range of therapeutic properties [33]. These properties make honey effective for treating a range of health conditions, including wounds, coughs and sore throats, digestive issues, skin conditions, immune system support, allergies, and cancer [20,21,22,23]. Raw, unpasteurized honey is considered to have the most therapeutic potential, as it retains its natural compounds, which can be destroyed through processing. With its multiple therapeutic applications, honey is a promising natural alternative to traditional pharmaceutical treatments [42]. Sidr honey has been used for medicinal purposes for centuries, particularly in traditional Arabic and Islamic medicine. It has potent antibacterial, anti-inflammatory, and antioxidant properties, making it effective in treating a range of health conditions. These include digestive issues, respiratory issues, wound healing, skin conditions, immune system support, and sexual health [29,43,44]. However, the therapeutic potential of Sidr honey may vary depending on factors such as the geographic location and environmental conditions in which the tree is grown [45].

The present research was conducted to investigate the anticancer activity of Saudi Sidr honey against a variety of cancer cells, including colorectal cancer cells (HCT-116), breast cancer cells (MCF-7), and lung cancer cells (A-549). Across all cancer cell lines studied, the Saudi Sidr honey exhibited cytotoxic effects dose-dependently. Based on the obtained IC_50_ values, HCT-116 cells were more susceptible to Saudi Sidr honey cytotoxic activity than other cancer cells. As a further measure of anticancer potential, colony formation assays were conducted to examine the honey’s antineoplastic properties. Several phytochemicals, such as flavonoids, phenolic acids, terpenoids, and others, are known to be present in the Saudi Sidr honey. It has been reported that some of these phytochemicals possess antineoplastic properties against various cancer cell lines. Flavonoids have been reported to possess antiproliferative properties against a wide range of cancers [46,47,48].

As part of our study, Annexin-V/PI flow cytometric assays were used to evaluate phosphatidylserine flipping as a possible pathway for cell death induced by Saudi Sidr honey. One of the biomarkers of apoptosis is the presence of phosphatidylserine at the exterior surface of the cell membrane [49]. The Annexin/PI assay also confirmed that Saudi Sidr honey was also able to induce apoptosis in cells. An important characteristic of necrosis is that it triggers an inflammatory response, which consequently causes collateral damage to normal cells in the surrounding microenvironment, unlike apoptosis, which does not trigger an inflammatory response [50]. In this way, apoptosis can be a protective mechanism that maintains tissue homeostasis by removing ailing and damaged cells [51]. The cells of cancer, on the other hand, are resistant to apoptosis for the purpose of sustaining their uncontrolled proliferation, and therefore, any compound that can modulate apoptosis would be a promising chemotherapeutic agent against cancer [52].

While the Saudi Sidr honey has antiproliferative properties, the cell cycle evaluation shows that Saudi Sidr honey restricts cells from progressing through G0/G1 phases, which is consistent with the antiproliferative activity. This suggests that Saudi Sidr honey perturbs the process of protein synthesis that is required for the progression of the cell from G1 to S-phase. Several studies have shown that the p53 and MDM2 proteins play an important role in the progression of the G0/G1 cell cycle [53,54]. A mechanism by which the Sidr honey plays a role in the disturbance of these proteins may be possible, but this aspect was not examined in this study. This may be due to the fact that the phytochemical constituents of the Saudi Sidr honey, such as flavonoids and phenolic acids, are believed to have an impact on cell cycle progression. Accordingly, the results presented here provide dependable evidence that Saudi Sidr honey contains promising compounds for treating colon cancer, which will be worth further investigation in the future to gain a deeper understanding of the compounds and to seek possible therapeutic applications.

There are some studies that have reported the anticancer activity of Sidr honey against different types of cancer cells. A study by Taha et al. (2015) [55] found that Sidr honey had anti-apoptotic effects on gastric tissues damaged by ethanol-induced ulceration. They also found that Sidr honey reduced the levels of inflammatory cytokines and prostaglandins in plasma. In addition, Ghramh et al. (2020) [56] reported that Sidr honey from three different sources (two from Saudi Arabia and one from Pakistan) demonstrated anticancer activity against liver (HepG2) cancer cells but not cervical (Hela) cancer cells. They also found that Sidr honey had antimicrobial, immunomodulatory, and silver nanoparticle production properties. A recent study conducted by Diebli et al. (2021) [57] found that Sidr honey from Algeria had anti-ulcerogenic and cytoprotective effects on gastric mucosa injured by a hydrochloric acid/ethanol mixture. They also found that Sidr honey modulated the oxidative stress and inflammatory response in the stomach. Bouali et al. (2023) [48] have recently found that Sidr honey derived from the Hail region had cytotoxic effects against lung (A549), breast (MCF-7), and colon (HCT-116) cancer cell lines, with IC_50_ values ranging from 5.203% to 7.257%. They also identified 10 phytochemical compounds and one tripeptide in Sidr honey that could be responsible for its antioxidant and anticancer activities. All of these studies, along with the results obtained from the present study, suggest that Sidr honey has potential anticancer properties that could be mediated by its phytochemical composition, antioxidant capacity, anti-inflammatory action, and modulation of apoptosis. However, more studies are needed to confirm the efficacy and safety of Sidr honey as a complementary or alternative therapy for cancer patients.

Furthermore, in the in silico analysis of the identified bioactive constituents of the Sidr honey with the target proteins of cancer, Palmidin C exhibited the highest binding affinities with all of the target proteins. There are not many studies that have directly investigated the anticancer activity of Palmidin C. However, a previous study conducted by Kushwaha et al., 2019 [58] reported for the first time, by means of a computational study, the anticancer potential of Palmidin C present in the *Bulbine frutescens* plant. It exhibited better potential to inhibit NF-kB, TGF-β, PI3K, and JAK2 proteins than their respective standard inhibitors. Therefore, Palmidin C is a novel potent anticancer chemical constituent identified from the present study that needs to be studied further in vitro and in vivo.

Moreover, in the present study, identification of the chemical constituents of the Saudi Sidr honey was carried out using GC–MS and HR-LCMS analysis without derivatization of the sample. Therefore, derivatization of the honey sample with commonly used derivatization agents, such as TMSiCl (trimethylsilyl chloride) and BSTFA (N,O-bis(trimethylsilyl)trifluoroacetamide), before analysis is recommended to enhance analyte volatility, stability, and sensitivity. These agents function to convert polar functional groups into less polar derivatives; hence, chromatographic behaviour and mass spectrometric detection are improved. The process broadens the range of compounds amenable to analysis, leading to the more accurate identification and quantification of diverse analytes in complex samples.

## 5. Conclusions

Overall, the results of this study indicate that Saudi Sidr honey is capable of inhibiting growth, causing apoptosis, and arresting the cell cycle in cancer cells. These findings are particularly compelling, as they indicate that the Saudi Sidr honey may contain bioactive compounds that exhibit anticancer properties and that should be isolated and characterized further.

## Figures and Tables

**Table 1:** Chemical components of Saudi Sidr honey by GC–MS chromatogram.

Compounds	Structure	RT (min)
Glyceraldehyde	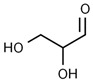	6.526
Butanedioic acid	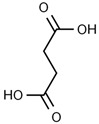	17.681
Tetrahydro-4H-pyran-4-ol	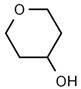	19.335
1-Cyclohexylimidazolidin-2-one	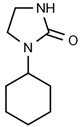	23.937
3-butyl-3-methyl-1-Cyclohexanone	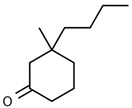	25.170
4-Butyl-3-methoxy-2-cyclopenten-1-one	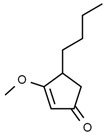	25.284
2,2,3,3-Tetramethylcyclopropanecarboxylic acid	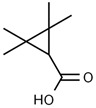	29.099
3,5-Dihydroxy-2-(3-methylbut-2-en-1-yl)	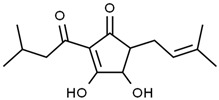	29.709

**Table 2:** List of chemical components identified from Saudi Sidr honey via HR-LCMS analysis.

Compounds	Mode of Analysis	Structure	RT (min)
Gerberinol	Positive	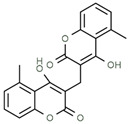	1.014
Osmaronin	Positive	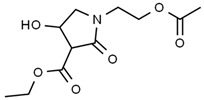	1.393
Retronecine	Positive	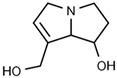	1.469
N-(1-Deoxy-1-fructosyl) phenylalanine	Positive	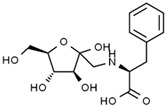	1.971
C16 Sphinganine	Positive	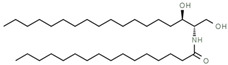	12.085
6-Hydroxypentadecanedioic acid	Positive	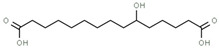	12.788
Sphinganine	Positive	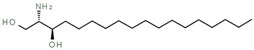	13.77
Oleamide	Positive	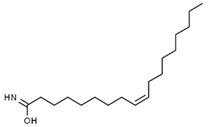	20.347
Hamamelose	Negative	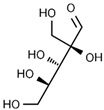	1.236
2-O-b-D-Galactopyranosyl-D-xylose	Negative	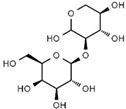	1.345
2,5-Didehydro-D-gluconate	Negative	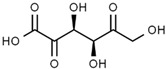	1.423
Sarmentosin epoxide	Negative	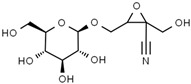	1.446
Hellicoside	Negative	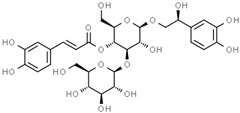	7.818
Palmidin C	Negative	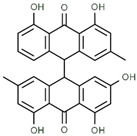	17.62
2-O-a-L-Fucopyranosyl-galactose	Negative	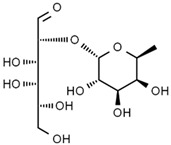	21.505

## Data Availability

All data that support the findings of this study are available within the article.
